# Continuous cursor tracking as a process-oriented measure of anticipation in soccer: expertise differences in spatial and temporal preparation

**DOI:** 10.3389/fspor.2026.1991767

**Published:** 2026-10-29

**Authors:** Osamu Nakamura, Ryoji Onagawa, Kazutoshi Kudo, Norikazu Hirose

**Affiliations:** 1 Laboratory of Sports Sciences, Department of Life Sciences, Graduate School of Arts and Sciences, The University of Tokyo, Tokyo, Japan; 2 Department of Psychology, Queen’s University, Kingston, ON, Canada; 3 Faculty of Sport Sciences, Waseda University, Tokyo, Japan

**Keywords:** anticipation, cursor tracking, decision-making, perceptual-cognitive expertise, preparatory state, soccer

## Abstract

**Introduction:**

Anticipating an opponent's actions is central to performance in team sports such as soccer, where a player must select the correct response within a fraction of a second. Perceptual-cognitive expertise in anticipation is well documented, but standard measures (response time, accuracy, or option choice) capture only the discrete response produced at a single moment and leave the preparatory processing that precedes it unobserved. Building on evidence that hand and cursor movements reveal cognition as it unfolds, the present study examined, as a proof of concept, whether continuous cursor tracking can read out the pre-response preparatory state in a sports anticipation task.

**Methods:**

Twelve skilled soccer players and twelve novices watched soccer pass-course clips and anticipated which teammate would receive the ball by moving a cursor; the video was stopped at the final frame (the moment a response was required), and the same clips were repeated across three blocks. Two continuous measures were defined: the cursor's Euclidean distance to the correct receiver at response onset (a spatial index of where the participant was poised) and the increase in cursor velocity in the window immediately before response onset (a temporal index of the movement toward the response).

**Results:**

Compared with novices, skilled players anticipated more accurately, were poised closer to the correct receiver, and showed a larger velocity increase; control analyses argued against the velocity difference being a by-product of the total amount of movement. In an exploratory analysis, the positional difference emerged across repeated presentations of the same clips and was largest in the final block. In addition, the velocity difference was already present in the first presentation, and no significant change across repeated presentations was detected.

**Discussion:**

The pre-response preparatory state is observed in both a spatial aspect (where one is poised) and a temporal aspect (how one moves toward the response). Because in basic reaching research the position and the velocity of movement index different aspects of decision-making, capturing both under a single continuous measure (which discrete responses cannot) provides a basis for measuring the decision-making processes that underlie anticipation in sport.

## 1 Introduction

In team sports such as soccer, players are repeatedly required to make accurate decisions under severe time constraints. In particular, the ability to anticipate an opponent's upcoming actions shapes the outcome of offensive and defensive play and substantially affects competitive performance (1, 2). In defensive situations, a player must anticipate within a fraction of a second which action—a pass, a shot, or a dribble—the attacking side will perform and toward whom, selecting from among several plausible options.

Athletes’ anticipation ability has typically been examined with the temporal occlusion paradigm, which originated in a study in which film of a tennis serve was occluded at different moments and observers predicted where the ball would land (3). In this paradigm, participants watch match footage and, immediately after the video is occluded, produce an anticipatory response indexed by response time, accuracy, or option evaluation. Using such discrete measures, skilled players have been shown to anticipate better than less-skilled players: they extract information from earlier advance cues (4) and, in soccer, outperform less-skilled players across multiple perceptual-cognitive skills (5) and in generating and prioritizing task-relevant options (6, 7). These findings are consistent across occlusion methods and between laboratory responses and interceptive actions (8). This expertise advantage has been consistently supported by meta-analytic and review evidence (9–11).

Although these measures capture the resulting response with high precision, they are obtained only once, at the moment a response is required. In actual anticipation, however, the information used changes over time, and the observer is thought to evaluate and narrow the candidate options before responding. The final response indicates which option was eventually selected, but not how the observer was prepared for it before the response was required—for example, whether the observer was already oriented toward one option or was still weighing several. Two observers who select the same option may therefore differ in how far, and how early, they had committed to it, and such differences cannot be recovered from accuracy or response time alone. Characterizing this pre-response preparatory state would thus complement the conventional measures, which describe the outcome of anticipation, by describing the process leading to that outcome. The present study targets this pre-response preparatory state.

One means of capturing the preparatory state is cursor tracking, which continuously records participants’ mouse or stylus movements while they make decisions (12, 13). Because the hand begins to move before a decision is finalized, the trajectory reflects how competing options are weighed over time (14, 15). This method has revealed moment-to-moment competition between candidate responses in diverse domains: trajectories were attracted toward a phonologically similar competitor when participants selected the picture named in a spoken instruction (16), curved toward the “yes” option when participants answered “no” to yes/no questions and fluctuated more when the questions were more uncertain (17), and deviated toward the truthful answer when participants were instructed to respond deceptively (18). Neurophysiological evidence further suggests that such trajectories reflect the relative activation of competing movement representations, which are encoded in parallel in the dorsal premotor cortex before a choice is made (19). Because the continuous trajectory can be observed without interruption up to the moment a response is required, the preparatory state can be read out from two aspects: the positional aspect—where the hand was poised at that moment—and the kinematic aspect—how it was being moved toward the response.

Both aspects are supported by research on reaching movements. Regarding the positional aspect, when participants were required to initiate rapid reaches toward one of several potential targets before the actual target was revealed, their initial trajectories were directed toward the spatial average of the potential targets and biased toward the side with more targets (20), and three-dimensional reach trajectories have since been proposed as a real-time probe of such competition (21). Regarding the kinematic aspect, movement dynamics have been shown to change with the confidence of the judgment and the state of competition: in the tasks described above, peak velocity was lower for questions with lower truth values (17) and was reached later during deceptive responses (18), and, more broadly, the vigor of a movement—the speed with which it is initiated and executed—scales with the subjective value of the chosen option (22). The former provides a basis for treating the hand position at the moment a response is required, and the latter for treating the increase in cursor velocity as that moment approaches, each as an observable index of the preparatory state. In the present study, these are framed as predictions at the behavioral level.

The present study used a soccer pass-course anticipation task. The video was stopped immediately before the pass and, without presenting the subsequent development, participants anticipated the correct receiver. This anticipation task was administered by repeating the same clips across multiple blocks. The pre-response preparatory state was operationalized using two measures defined relative to response onset (the moment the video stopped). The first measure was the proximity of the cursor to the correct receiver at response onset (the distance to the correct receiver), which represents how closely the observer was poised toward the correct receiver in a scene where the correct receiver was not indicated in the video. Because both groups viewed the same clips, the between-group comparison holds independently of differences in spatial layout. The second measure was the velocity increase of the cursor in the window immediately before response onset, which represents how much cursor velocity increased as the moment requiring a response approached. The former captures the position at which the participant was poised at the moment a response was required, and the latter the temporal change in the movement toward the response; the two constitute complementary aspects. In addition, after the anticipation task, a confidence rating task using the same pre-pass still images was administered to reinforce the interpretation from a perspective that does not depend on movement.

Based on the above, we made the following predictions. First, the skilled group would anticipate more accurately than the novice group. Second, at response onset, the skilled group would be poised closer to the correct receiver. Third, immediately before response onset, the skilled group would show a larger cursor velocity increase. Fourth, independently obtained confidence ratings were examined as a complementary, movement-independent measure expected to show a group-level difference consistent with those observed for the preparatory-state measures. These are positioned as observable behavioral predictions rather than predictions about underlying mechanisms. In addition, given that skilled players are superior not only in anticipating but also in recognizing previously seen patterns of play (23), that both the perception of structured patterns between players and advance postural cues contribute to anticipation (24), and that the relative contribution of these perceptual-cognitive skills varies with task constraints (25), we examined, in an exploratory manner, how these preparatory-state measures change across repeated presentations.

## 2 Materials and methods

### 2.1 Participants

Twenty-four male Japanese university students (age: *M* = 20.4 years, *SD* = 2.0) participated in this study. All participants had normal or corrected-to-normal vision. The skilled group (*n* = 12; age: *M* = 19.1 years, *SD* = 1.1) comprised players belonging to Division 1 of the Kanto Men's University Soccer League, with soccer experience of *M* = 12.9 years (*SD* = 2.1) and team practice of approximately 2 h per day, 6 days per week. The novice group (*n* = 12; age: *M* = 21.7 years, *SD* = 1.8) consisted of students with no competitive soccer experience. The novice group included participants with basic experience in sports such as baseball (*n* = 6), tennis (*n* = 2), track and field (*n* = 2), and sumo (*n* = 2), but none had experience in sports with perceptual-motor demands similar to those of soccer. Participants were unaware of the purpose of the study and provided written informed consent. This study was approved by the Ethics Committee of the Graduate School of Sport Sciences at Waseda University, and all experimental procedures were conducted in accordance with the approved guidelines.

### 2.2 Experimental setup

Participants were seated in a quiet, dim room. A pen tablet (Wacom, Intuos 4 Extra Large; workspace, 488 × 305 mm) was placed on a desk to record arm movements. A monitor (I-O DATA, KH2500V-ZX2, 24.5 inches, 1920 × 1080 pixels, a vertical refresh rate of 240 Hz) was placed vertically above the pen tablet, positioned so that its center was aligned with the participant's eye level at a viewing distance of approximately 1.0 m (Figure 1A). The position of the stylus on the pen tablet was mapped in real time to a cursor on the display. Cursor positions were recorded at 30 Hz. Stimulus presentation and data collection were implemented in MATLAB (MathWorks, Natick, MA, USA) using the Psychophysics Toolbox.

**Figure 1:**
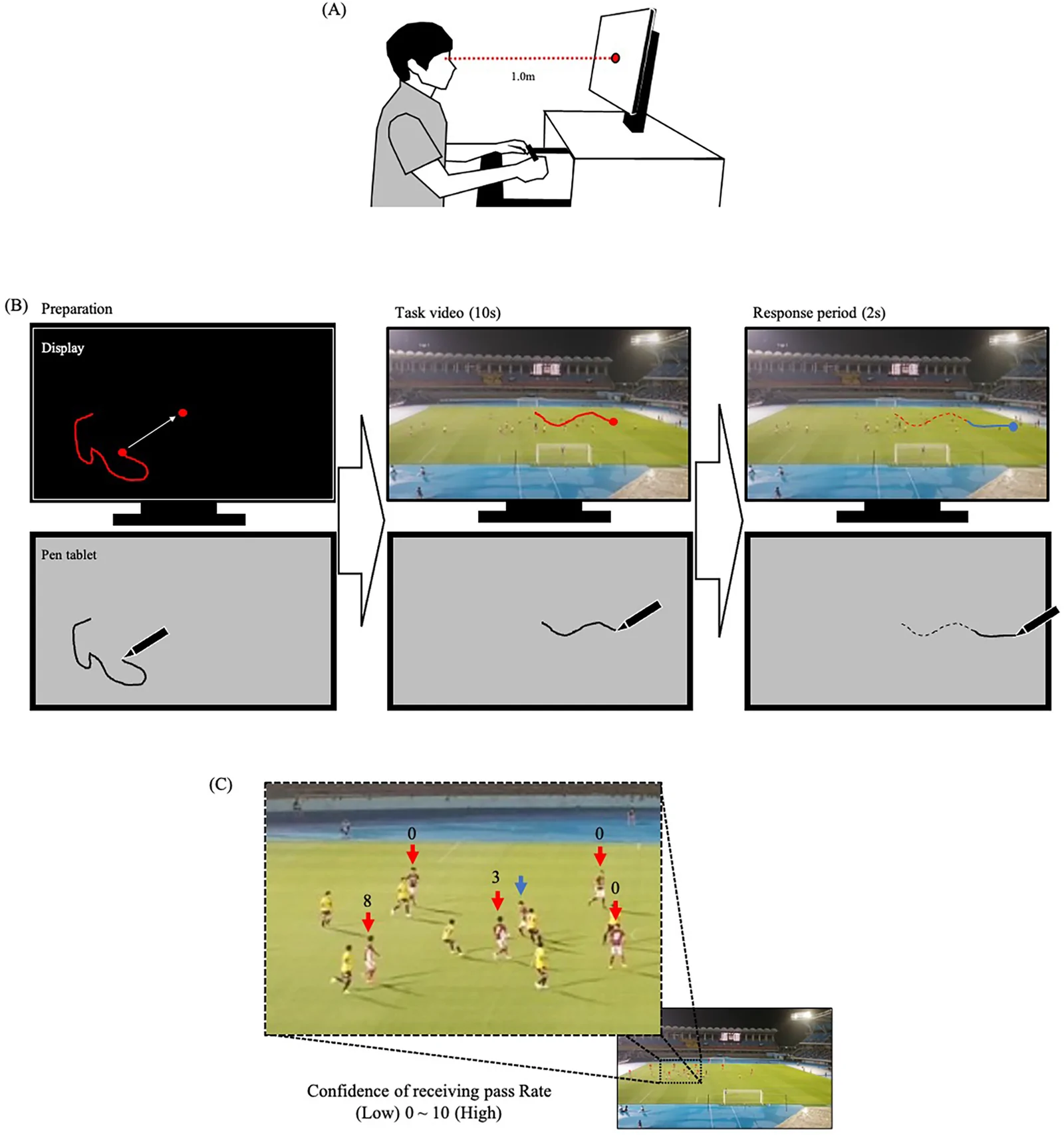
Experimental setup and task procedure. **(A)** Experimental setup. **(B)** Anticipation task. One red dot is fixed in the center, and another is synchronized with the cursor. Hover the cursor over the center point to start the experimental stimuli. 10-second video of the task: The cursor is indicated by a red dot. 2-second still image (response period): The cursor is indicated by a blue dot. **(C)** Confidence rating task. Blue arrows indicate a final ball passer, and red arrows indicate the other 10 offensive players.

### 2.3 Tasks and procedure

#### 2.3.1 Pass-course anticipation task

The anticipation task used videos from matches in the Kanto University Soccer League. The videos were recorded with a Galaxy S8 (resolution 1920 × 1080, frame rate 30 Hz) to provide a bird's-eye view of all 22 players on the field. In each clip, the attacking team advances from the top of the screen by passing or dribbling the ball. This top-down view has frequently been used in previous studies to assess anticipation skill in competitive sports (6, 7). Twenty clips were selected to provide a variety of distances between the receiver and the passer. The final frame of each video was the moment the passer planted their standing foot immediately before making a pass. Each video was 10 s long.

At the beginning of each trial, a black screen with two red dots was presented. One dot was fixed at the center of the screen, and the other was controlled by the stylus position. The video began when the participant overlapped the two dots. Participants were free to move the cursor during the video and were instructed to predict which player would receive the pass. After the video ended, the final frame was presented as a still image for 2 s, during which participants were required to move the cursor to the predicted receiver and complete the reach within the response period (Figure 1B).

Participants first completed 10 familiarization trials, followed by 60 experimental trials (20 clips  ×  3 blocks) presented in random order. Participants were not informed that the same videos would be repeated across blocks; that is, each participant viewed each clip three times (three blocks).

#### 2.3.2 Confidence rating task

Participants were presented with 20 still images, each corresponding to the final frame of a clip used in the anticipation task. For each image, participants were instructed to rate, on a scale from 0 (*not at all likely*) to 10 (*very likely*), the likelihood that each of the passer's teammates would receive the ball. In the images, the ball passer was indicated by a blue arrow and the 10 offensive teammates by red arrows. Participants wrote their confidence ratings directly on the red arrows representing the potential receivers. No time limit was set for this task (Figure 1C).

#### 2.3.3 Procedure

Before the experiment, participants were given an overview of the experimental procedure and asked to complete a consent form if they agreed to participate. They also completed a questionnaire on their age, vision, and athletic history from elementary school to university (sports played, years of competition, practice frequency, and competitive performance). After the questionnaire, participants completed the anticipation task and then the confidence rating task, in that order. The anticipation task and the confidence rating task each lasted approximately 20 min, with a break between them, and the entire session, including the explanation and preparation, lasted approximately 1 h.

### 2.4 Data processing and outcome measures

One of the 20 clips was excluded from analysis because its duration was longer than the others and caused technical problems. Consequently, 57 trials (19 clips  ×  3 blocks) were analyzed in the anticipation task and 19 trials in the confidence rating task. For each trial, the torso center of each offensive player was manually identified, and the player who ultimately received the pass was defined as the correct receiver.

The pre-response preparatory state was quantified using two measures defined relative to response onset (the moment the video stopped). Response onset was the moment at which the video stopped and a response was required of the participant, corresponding to the preparatory state immediately before committing to a response.

The distance to the correct receiver was defined as the Euclidean distance (px) between the cursor coordinates and the final receiver's coordinates at response onset (Figure 2A). A smaller value indicates that, at the moment a response was required, the participant was poised closer to the correct receiver. Anticipation accuracy was computed for each participant as the percentage of correct trials out of 57, where a correct trial was one in which the Euclidean distance between the cursor coordinates and the final receiver's coordinates at the end of the response period was within 50 px. This criterion was set relative to the spacing between players: the nearest other attacking player was at least 55 px (median 118 px) from the correct receiver, and no other attacker was within 50 px of the correct receiver in any clip.

**Figure 2:**
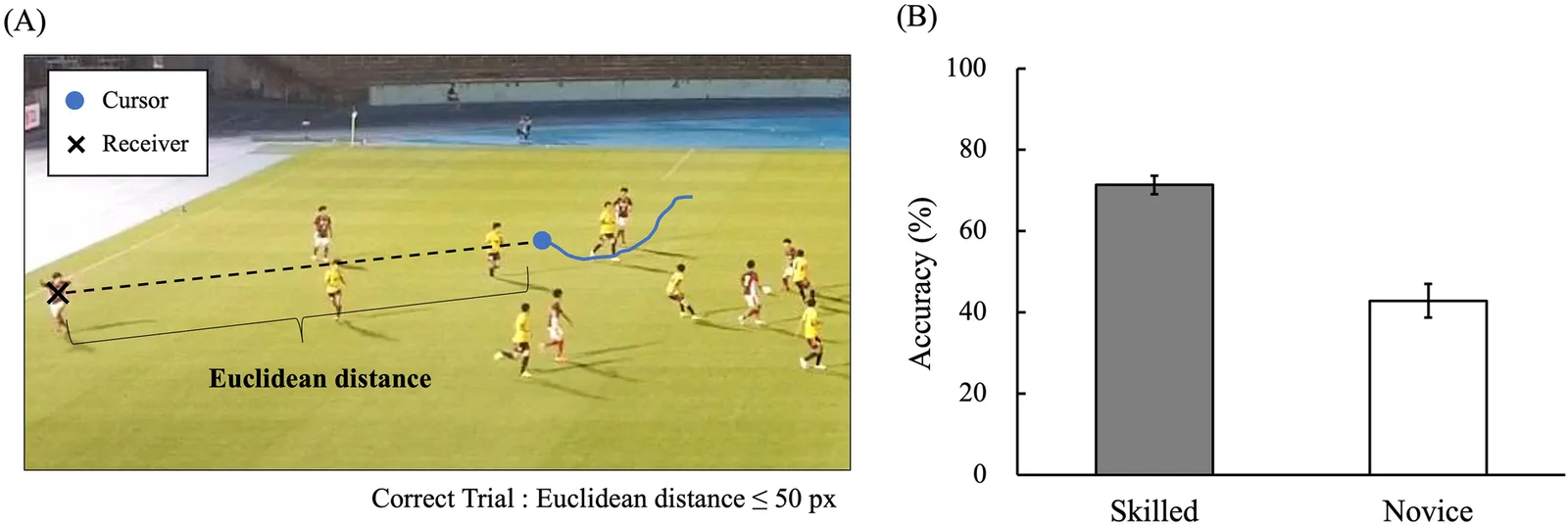
Evaluation metrics and results for anticipation accuracy. **(A)** Calculation of the Euclidean distance used to determine accuracy, computed at the end of the response period. Blue dot: coordinates of the cursor. Black cross: coordinates of the correct receiver. **(B)** Comparison of the anticipation accuracy. Each bar represents the between-participant mean, and the error bars represent the standard errors.

Cursor velocity was computed from the recorded cursor position. To attenuate high-frequency noise, the two-dimensional cursor position was low-pass filtered with a second-order Butterworth filter (cutoff frequency = 4 Hz) applied in both the forward and reverse directions to achieve zero-phase filtering, and frame-by-frame velocity (px/s) was then obtained by forward differencing of the filtered position. All velocity-based measures (the velocity increase and the peak velocity after response onset) were derived from this filtered velocity. The velocity increase was defined as the slope (px/s^2^) obtained by fitting, within a time window preceding response onset, a least-squares linear regression of frame-by-frame cursor velocity (px/s) on relative time (s), with response onset set to 0. The regression was fitted separately for each trial, and the resulting trial-wise slopes were averaged within each participant to obtain a participant-level value. A positive value indicates that cursor velocity increased as the moment requiring a response approached. The window length for the main analysis was 1.5 s, a duration common to all clips, because defining intervals on an event basis for each clip would cause the interval length to vary across videos and produce non-uniform computation conditions for movement measures that depend on interval length. The specific value of a 1.5-s window was not essential to the conclusions; sensitivity analyses varying the window length across 1.0, 1.5, and 2.0 s were also conducted, confirming that the results did not depend on the choice of window length.

To control for the amount of movement, the following measures were also computed: peak velocity after response onset (the maximum cursor velocity during the movement phase after the video stopped), movement amplitude (the straight-line distance from the cursor position at response onset to the final cursor position), and total trajectory length (the sum of cursor displacement across the entire viewing period).

The confidence rating was defined, for each participant, as the mean confidence assigned to the correct receiver.

### 2.5 Statistical analysis

Statistical analyses were performed in R using the afex, effectsize, and BayesFactor packages, and the significance level was set at .05 for all tests. The between-group difference (skilled vs. novice) was treated as a confirmatory analysis specified *a priori*, and the pattern of change across repeated presentations (blocks) as an exploratory analysis. For anticipation accuracy, the distance to the correct receiver, and the velocity increase, a mixed-design ANOVA with group as a between-subjects factor and block as a within-subjects factor was computed with the afex package (Type III sums of squares); the main effect of group was reported as a confirmatory finding, and the group  ×  block interaction and the simple main effects of block within each group as exploratory findings. For within-subjects effects, sphericity was assessed with Mauchly's test; because this test may have limited power to detect violations of sphericity in small samples, the Huynh–Feldt correction was applied to all within-subjects effects irrespective of its outcome. Between-group differences for the whole-sample measures (anticipation accuracy, peak velocity, movement amplitude, total trajectory length, and the confidence rating) were tested with independent-samples (Student's) *t* tests, and the block-wise between-group differences with Welch's *t* tests. Effect sizes were obtained with the effectsize package—Cohen's *d* for *t* tests and *η*p^2^ for ANOVA effects—each with its 95% confidence interval. To examine whether the group difference in velocity increase was attributable to movement magnitude, an ANCOVA was conducted with movement amplitude as a covariate, and the homogeneity-of-slopes assumption was assessed with the movement amplitude  ×  group interaction. As a sensitivity analysis, the velocity increase was recomputed with the pre-onset window set to 1.0, 1.5, and 2.0 s, and the main effect of group was re-evaluated for each window. In addition to the frequentist tests, Bayes factors (BF₁₀) were computed with the BayesFactor package (JZS prior) to quantify the strength of evidence for each effect.

## 3 Results

### 3.1 Anticipation accuracy

We first examined anticipation accuracy to test whether the skilled and novice groups differed in overall anticipation performance. The skilled group showed a higher percentage of correct responses than the novice group (Skilled: *M* = 71.64%, *SD* = 8.49; Novice: *M* = 43.13%, *SD* = 15.10). The between-group difference was significant, with a large effect size, *t*(22) = 5.70, *p* < .001, *d* = 2.33, 95% CI [1.26, 3.36], BF₁₀ = 1490 (Figure 2B). This result indicates that the preparatory-state measures examined below were obtained in a context accompanied by a clear difference in anticipation ability. Across blocks, accuracy increased in both groups (skilled: 68.4%, 70.6%, 75.9%; novice: 39.0%, 43.0%, 47.4%), main effect of block, *F*(2, 44) = 4.23, *p* = .021, *η*p^2^ = .16, 95% CI [.02, 1.00], BF_10_ = 2.61, with no group  ×  block interaction, *F*(2, 44) = 0.05, *p* = .950, *η*p^2^ = .00, 95% CI [.00, 1.00], BF_10_ = 0.20.

### 3.2 Preparatory state at response onset: distance to the correct receiver

To assess the spatial aspect of the pre-response preparatory state, we examined the distance of the cursor to the correct receiver at response onset; a smaller value indicates that, at the moment a response was required, the observer was poised closer to the correct receiver. The main effect of group was significant, with the skilled group poised closer to the correct receiver than the novice group (Skilled: *M* = 315.58 px, *SD* = 39.89; Novice: *M* = 347.97 px, *SD* = 29.70), *F*(1, 22) = 5.09, *p* = .034, *η*p^2^ = .19, 95% CI [.01, 1.00], BF₁₀ = 2.24.

As an exploratory analysis, changes across repeated presentations were examined. A group  ×  block interaction was found, *F*(1.96, 43.17) = 4.88, *p* = .013, *η*p^2^ = .18, 95% CI [.03, 1.00], BF₁₀ = 4.67. In the skilled group, the distance decreased across blocks (simple main effect of block, *F*(2, 22) = 9.94, *p* = .001, *η*p^2^ = .47, 95% CI [.19, 1.00], BF₁₀ = 37.7), such that the skilled group became poised closer to the correct receiver with repeated presentation. In the novice group, by contrast, the distance was approximately constant across blocks, *F*(1.65, 18.10) = 0.12, *p* = .846, *η*p^2^ = .01, 95% CI [.00, 1.00], BF₁₀ = 0.20. The between-group difference within each block was marginal in Block 1 (*t*(16.43) = −1.84, *p* = .084, *d* = −0.75, 95% CI [−1.58, 0.10], BF₁₀ = 1.22), nonsignificant in Block 2 (*t*(21.22) = −1.04, *p* = .308, *d* = −0.43, 95% CI [−1.23, 0.39], BF₁₀ = 0.55), and significant and largest in Block 3 (*t*(21.40) = −2.95, *p* = .008, *d* = −1.20, 95% CI [−2.07, −0.31], BF₁₀ = 6.42; Figure 3A). Because both groups viewed the same clips, this group difference is independent of the spatial layout of individual videos. In summary, the skilled group was poised closer to the correct receiver at response onset, and this positional difference emerged across repeated presentations and was largest in the final block.

**Figure 3:**
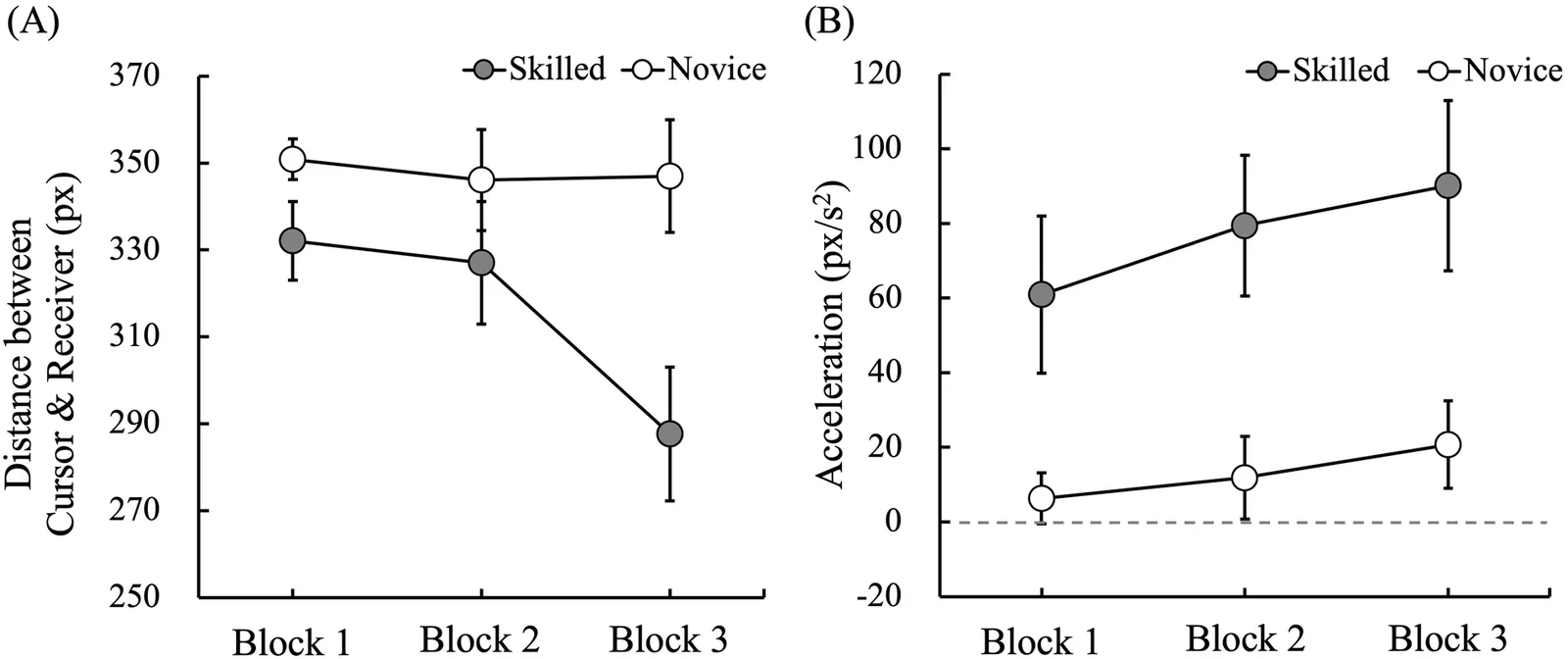
Results for preparatory state in the anticipation task. **(A)** The Euclidean distance of the cursor to the correct receiver at response onset. **(B)** The cursor velocity increase in the window immediately before response onset (1.5 s). Each dot represents the between-participant mean, and the error bars represent the standard errors.

### 3.3 Preparatory state immediately before response onset: velocity increase

To assess the kinematic aspect of the pre-response preparatory state, we examined the velocity increase in the window immediately before response onset, together with control measures for the amount of movement. In a 1.5-s window before response onset, the slope of cursor velocity regressed on time was computed as the velocity increase. A positive value indicates that cursor velocity increased as the moment requiring a response approached. The main effect of group was significant, with the skilled group showing a larger velocity increase than the novice group (Skilled: *M* = 75.69 px/s^2^, *SD* = 61.68; Novice: *M* = 11.75 px/s^2^, *SD* = 27.91), *F*(1, 22) = 10.71, *p* = .003, *η*p^2^ = .33, 95% CI [.08, 1.00], BF₁₀ = 11.6. The between-group difference was significant in every block (Block 1: *t*(13.35) = 2.50, *p* = .026, *d* = 1.02, 95% CI [0.12, 1.89], BF₁₀ = 3.08; Block 2: *t*(17.92) = 3.13, *p* = .006, *d* = 1.28, 95% CI [0.36, 2.16], BF₁₀ = 8.77; Block 3: *t*(16.73) = 2.74, *p* = .014, *d* = 1.12, 95% CI [0.22, 1.99], BF₁₀ = 4.51).

In the exploratory analysis, and unlike the positional measure, no change was found across repeated presentations. Mauchly's test indicated a violation of sphericity for the velocity increase (*W* = 0.69, *p* = .022; Huynh–Feldt *ɛ* = .81). Neither the group  ×  block interaction, *F*(1.62, 35.73) = 0.30, *p* = .696, *η*p^2^ = .01, 95% CI [.00, 1.00], BF₁₀ = 0.24, nor the main effect of block, *F*(1.62, 35.73) = 2.13, *p* = .142, was significant, and the group difference in velocity increase was present from the first presentation and in every block (Figure 3B). This group difference was consistently observed even when the analysis window was varied from 1.0 to 2.0 s (main effect of group, *F* = 6.40–14.64, all *p* < .05).

We examined whether the group difference in velocity increase was a by-product of movement magnitude arising because skilled players moved to more distant locations. During the phase in which the cursor moved to the final predicted location after the video stopped, there was no group difference in peak velocity (Skilled: *M* = 755.58 px/s, *SD* = 193.56; Novice: *M* = 702.01 px/s, *SD* = 218.68), *t*(22) = 0.64, *p* = .532, *d* = 0.26, 95% CI [−0.55, 1.06], BF₁₀ = 0.43. Nor was there a significant group difference in movement amplitude from response onset to the final location (Skilled: *M* = 270.32 px, *SD* = 54.69; Novice: *M* = 233.83 px, *SD* = 68.45), *t*(22) = 1.44, *p* = .163, *d* = 0.59, 95% CI [−0.24, 1.40], BF₁₀ = 0.78. Examining the relationship between velocity increase and movement amplitude within each group, the homogeneity-of-slopes assumption required for a conventional ANCOVA was not met (movement amplitude  ×  group interaction, *F*(1, 20) = 5.90, *p* = .025): the slope was negative in the skilled group (*b* = −0.78) and near zero in the novice group (*b* = −0.12). This pattern is itself inconsistent with a movement-magnitude account, under which a larger amount of movement would be expected to accompany a larger velocity increase; within the skilled group, a larger movement amplitude was instead associated with a smaller velocity increase. The response-onset-aligned time series of cursor velocity showed a two-phase structure: the skilled group accelerated more during the preparatory phase before the video stopped, whereas the peaks of the two groups coincided during the movement phase after the stop. Total trajectory length, which represents the total amount of cursor movement, tended to be greater in the novice than in the skilled group rather than smaller (Skilled: *M* = 1005.11 px, *SD* = 253.57; Novice: *M* = 1250.19 px, *SD* = 379.81), *t*(22) = −1.86, *p* = .076, *d* = −0.76, 95% CI [−1.58, 0.08], BF₁₀ = 1.25 — the opposite of what a movement-magnitude account would predict. In summary, the skilled group showed a larger pre-response velocity increase than novices from the first presentation onward, and this difference was not accounted for by the amount of movement.

### 3.4 Group difference in the confidence rating

To examine whether the group difference also appeared in a measure without a time constraint, we compared the confidence rating for the correct receiver between groups. After the anticipation task, a confidence rating task was conducted using the same pre-pass still images as in the anticipation task. The skilled group assigned higher confidence to the correct receiver than the novice group, *t*(22) = 3.21, *p* = .004, *d* = 1.31, 95% CI [0.41, 2.19], BF₁₀ = 10.2. Whereas the pass-course anticipation task required a response within 2 s of the stop, the confidence rating task imposed no time limit. In summary, the skilled group assigned higher confidence to the correct receiver than novices, showing a group difference in the same direction as in the anticipation task under a task without a time constraint.

## 4 Discussion

### 4.1 Summary of results

The present study examined, as a proof of concept, whether cursor tracking can serve as a means of measuring the pre-response preparatory state in a sports anticipation task. The skilled group anticipated more accurately than the novice group (percentage correct, *p* < .001, *d* = 2.33). For the two preparatory-state measures, the distance to the correct receiver at response onset showed a main effect of group (*p* = .034, *η*p^2^ = .19) and a group  ×  block interaction (*p* = .013, *η*p^2^ = .18), and the velocity increase immediately before response onset showed a main effect of group (*p* = .003, *η*p^2^ = .33). In what follows, we discuss in turn what these findings establish about the present method as a measurement tool and how the method might be extended from there.

### 4.2 Expertise differences in the anticipatory preparatory state (confirmatory findings)

The present study read out, from the continuous cursor trajectory, this preparatory state that is not obtained from discrete responses, along two aspects: the position at which the participant was poised at the moment a response was required (the distance to the correct receiver) and the temporal change in the movement toward the response (the velocity increase). As predicted *a priori*, an expertise difference was found in both. The skilled group was poised closer to the correct receiver at response onset and increased cursor velocity more immediately before responding. The Bayesian evidence for the positional difference was, however, weaker than that for the velocity difference.

The group difference in the position of poise does not derive from the spatial layout of individual videos. Both groups viewed the same clips, and the position of the correct receiver was common across groups. This group difference is therefore observed as a difference in preparation that is independent of the video layout.

Several analyses argue against the possibility that the group difference in velocity increase was a by-product of movement magnitude arising because skilled players moved to more distant locations. In the phase in which the cursor moved to the final predicted location after the video stopped, there was no group difference in peak velocity, and the two groups did not differ significantly in movement amplitude. Examining the relationship between velocity increase and movement amplitude within each group, however, the homogeneity-of-slopes assumption required for a conventional ANCOVA was not met: the slope was negative in the skilled group and near zero in the novice group. This slope pattern is itself inconsistent with a movement-magnitude account, because within the skilled group a larger movement amplitude was associated with a smaller, not a larger, velocity increase. Consistent with this, total trajectory length tended to be greater in the novice than in the skilled group, again the opposite of what such a confound would predict. The response-onset-aligned velocity time series showed a two-phase structure, in which the skilled group accelerated more during the preparatory phase before the stop, whereas the peaks of the two groups coincided during the movement phase after the stop. Together, these results indicate that what differs between groups is not the total amount of movement but the position of poise and the temporal change in movement immediately before the response themselves.

The larger velocity increase in the skilled group may also reflect greater confidence in, or earlier commitment to, the anticipated response. Movement dynamics have been shown to vary with the confidence of the judgment (17), and movement vigor increases with the subjective value of the chosen option (22). Consistent with this possibility, the skilled group assigned higher confidence to the correct receiver in the confidence rating task. Because the peak velocity after response onset did not differ between groups, such confidence may have been expressed as greater acceleration before response onset rather than as a faster movement after it, which may relate to the shorter response times commonly reported for skilled performers (11). However, because confidence was not measured on a trial-by-trial basis in the anticipation task, this interpretation remains to be tested.

### 4.3 Dissociation of the two aspects across repeated presentations (exploratory findings)

How these preparatory-state measures change across repeated presentations of the same scene was examined in an exploratory manner.

For the distance to the correct receiver at response onset, a group  ×  block interaction was found. The skilled group reduced this distance across repeated presentations, whereas the poise of the novice group was approximately constant across repetitions. As a result, the group difference depended on repeated presentation and was largest in the final block. In Block 3, this difference (59.4 px) corresponded to 1.19 times the 50-px accuracy criterion; because both groups were still roughly 290–350 px from the correct receiver at response onset, it reflects the relative position of poise before the response rather than a near-final selection. This change suggests that the preparatory state is not fixed but can be updated in response to repeated presentation of the same scene. As background, two accounts are consistent with this: skilled players may have extracted clip-specific structure and anticipated earlier (23–25), and repeated presentation may have led participants to memorize the location they had selected on previous presentations and to become poised toward it in anticipation (26). Because the correct receiver was never shown to the participants, the latter account concerns memory for one's own previous selection rather than for the correct answer. If such memory alone were responsible, a comparable change would be expected in both groups; the finding that the change was limited to the skilled group is therefore more directly compatible with a more efficient extraction or use of clip-specific structure by skilled players. Nevertheless, memory-based poise could also depend on expertise if recognizing a scene as previously seen is itself facilitated by expertise (23), and such recognition may have required more than one previous presentation, which could account for the change appearing in the final block. The present design does not separate the two.

By contrast, the group difference in velocity increase was significant from the first presentation and in every block, with no significant group  ×  block interaction. This group difference was consistently observed when the analysis window was varied from 1.0 to 2.0 s. However, given the limited sensitivity of the present sample to small changes, the absence of a significant interaction does not establish that kinematic preparation is invariant across repeated presentations.

Taken together, a group  ×  block interaction was detected for the positional measure but not for the kinematic measure. This pattern is consistent with the two aspects being at least partly separable, but the present design cannot determine whether they are complementary aspects of a single preparatory state or two separate processes. Even so, the present method allows both aspects to be observed with a single continuous measure, which is not possible with discrete end-point responses.

### 4.4 Limitations

The findings of the present study were obtained under several conditions. With 12 participants per group contrasting two clearly separated groups, the present study is best regarded as a proof of concept. Because the between-group effects reported here were relatively large, they were detectable at this sample size; nonetheless, sensitivity to smaller effects may have been limited, and the findings should be interpreted with appropriate caution regarding statistical power. Replication in larger and more diverse samples, spanning a more continuous range of skill levels, would help establish the robustness and generality of the present findings. The number of stimulus clips was also limited. The videos were not corrected for perspective, so a given pixel distance does not correspond to the same field distance across the image; because both groups viewed identical frames in every block, however, any distortion affects the between-group and between-block comparisons equally. The changes across repeated presentations were examined in an exploratory manner; because the between-group test within each block for the positional measure included marginal significance, its interpretation is not definitive, and we relied on the main effect of group and the interaction. The extraction of clip structure and the anticipatory poise mediated by memory of previously selected locations, offered as background, cannot be separated by the present design. The sources of information used for anticipation (contextual cues, kinematic information, prior information) were not manipulated and remain for future examination.

### 4.5 Potential for development in sports anticipation research

Conventional measures of anticipation, such as response accuracy, response time, and confidence ratings, are obtained only after the decision has been made. In the present study, the two groups differed clearly in anticipation accuracy, the measure of the final response, but two further differences were observed only before the response: the peak cursor velocity after the video stopped did not differ significantly between groups, whereas the velocity increase before response onset did; and anticipation accuracy improved to a similar extent in the two groups across repeated presentations, whereas the position of poise at response onset changed only in the skilled group. The preparatory-state measures thus revealed group differences for which no counterpart was detected in the corresponding measures of the subsequent response. This property may be relevant to issues highlighted for future research on anticipation, such as how contextual information is integrated with emerging kinematic information as an action unfolds and how athletes respond to deceptive actions (10), because these issues concern how the observer's preparation changes before the response rather than the response itself.

The framework of the present study—reading out the pre-response preparatory state from the cursor trajectory—derives from basic research on reaching movements, in which position and velocity have each been characterized as indices reflecting different cognitive aspects of decision-making. Although the present study did not measure these decision-making processes themselves, each of the two aspects suggests a direction for developing the method in the context of sports anticipation.

First, in basic research, position has been shown to reflect the competition among candidates, and that competition is read out on the movement trajectory (19). Although the positional measure in the present study is confined to the position at a single time point at the moment a response is required and does not analyze the shape of the trajectory, using the same continuous measure could extend the analysis to tracking, from the shape of the trajectory, how the competition among candidates is resolved (27), or to examine changes of mind and hesitation (28, 29) during anticipation, for example when an observer responds to a deceptive action.

Second, in basic research, velocity has been shown to reflect the decision-making process over time (22). The two-phase structure of preparation and execution shown by the velocity time series in the present study indicates that the method can decompose the temporal structure of the movement toward the response, and provides a foothold for analyses that track the temporal dynamics of decision-making in this domain.

Furthermore, the possible dissociation in the dynamics that position and movement showed across repeated presentations raises a mechanistic question that the present design cannot answer. Whether the updating of the spatial poise and the stability of the kinematic preparation derive from different processes can be examined with a design that manipulates the two independently.

These directions follow from the descriptive findings of the present study and indicate how an established method can be extended to the measurement of decision-making processes in this domain and, potentially, in other domains. The value of the present study as a proof of concept lies in its presentation of a basis on which such questions can be addressed under a single behavioral measure.

## 5 Conclusion

This study provides a proof of concept that cursor tracking can measure the pre-response preparatory state in a sports anticipation task. Skilled players were poised closer to the correct receiver at response onset and increased cursor velocity more immediately before responding, and these differences were independent of the total amount of movement. The positional and kinematic aspects showed different patterns of change across repeated presentations: the positional difference amplified with repetition, whereas the velocity difference was present from the first presentation. Under a single continuous behavioral measure, the method reads out the preparatory state from complementary spatial and temporal aspects that discrete responses do not capture. This provides a basis on which questions about the decision-making processes underlying sports anticipation can be addressed.

## Data Availability

The raw data supporting the conclusions of this article will be made available by the authors, without undue reservation.
